# Implementing Legislation to Improve Hospital Support of Breastfeeding, New York State, 2009–2013

**DOI:** 10.5888/pcd12.150121

**Published:** 2015-07-30

**Authors:** Barbara A. Dennison, Bethany A. Hawke, Rachael A. Ruberto, Deborah J. Gregg

**Affiliations:** Author Affiliations: Bethany A. Hawke, medical student, Albany Medical School, Albany, New York. Rachael A. Ruberto, Deborah J. Gregg, New York State Department of Health, Albany, New York. Dr. Dennison is also affiliated with the School of Public Health, University at Albany–State University of New York, Rensselaer, New York.

## Abstract

**Introduction:**

Increasing breastfeeding is a public health priority supported by strong evidence. In 2009, New York passed Public Health Law § 2505–a, requiring that hospitals support the World Health Organization’s (WHO’s) recommended “Ten Steps for Successful Breastfeeding” (Ten Steps). This legislation strengthened and codified existing New York State’s hospital perinatal regulations. The purpose of this study was to assess hospital policy compliance with New York laws and regulations related to breastfeeding.

**Methods:**

In 2009, 2011, and 2013, we collected written breastfeeding policies from 129 New York hospitals that provided maternity services. A policy review tool was developed to quantify compliance with the 28 components of breastfeeding support specified in New York Codes, Rules, and Regulations and the new legislation. In 2010 and 2012, hospitals received individual feedback from the New York State Department of Health, which informed hospitals in 2012 that formal regulatory enforcement, including potential fines, would be implemented for noncompliance.

**Results:**

The number of components included in hospital policies increased from a mean of 10.4 in 2009, to 16.8 in 2011, and to 27.1 in 2013) (*P* < .001); a greater increase occurred from 2011 through 2013 than from 2009 through 2011 (*P* < .001). The percentage of hospitals with fully compliant policies increased from 0% in 2009, to 5% in 2011, and to 75% in 2013 (*P* < .001), and the percentage that included all WHO’s 10 steps increased from 0% to 9% to 87%, respectively (*P* < .001).

**Conclusion:**

Although legislation or regulations requiring certain practices are important, monitoring with enforcement accelerates, and may be necessary for, full implementation. Future research is needed to evaluate the impact of improved hospital breastfeeding policies on breastfeeding outcomes in New York.

## MEDSCAPE CME

Medscape, LLC is pleased to provide online continuing medical education (CME) for this journal article, allowing clinicians the opportunity to earn CME credit.

This activity has been planned and implemented in accordance with the Essential Areas and policies of the Accreditation Council for Continuing Medical Education through the joint sponsorship of Medscape, LLC and *Preventing Chronic Disease*. Medscape, LLC is accredited by the ACCME to provide continuing medical education for physicians.

Medscape, LLC designates this Journal-based CME activity for a maximum of 1 ***AMA PRA Category 1 Credit(s)™***. Physicians should claim only the credit commensurate with the extent of their participation in the activity.

All other clinicians completing this activity will be issued a certificate of participation. To participate in this journal CME activity: (1) review the learning objectives and author disclosures; (2) study the education content; (3) take the post-test with a 75% minimum passing score and complete the evaluation at www.medscape.org/journal/pcd; (4) view/print certificate.

**Release date: July 30, 2015; Expiration date: July 30, 2016**

### Learning Objectives

Upon completion of this activity, participants will be able to:

1. Discuss the regulation of hospitals in New York regarding breastfeeding protocols
2. Assess adherence to breastfeeding protocols in New York before the introduction of regulatory changes
3. Evaluate the effects of legislation and regulatory oversight on breastfeeding promotion in New York
4. Distinguish particularly effective public tools that improved breastfeeding promotion in hospitals in New York

**EDITORS**

Rosemarie Perrin, Editor, *Preventing Chronic Disease*. Disclosure: Rosemarie Perrin has disclosed no relevant financial relationships.

**CME AUTHOR**

Charles P. Vega, MD, Clinical Professor of Family Medicine, University of California, Irvine

Disclosure: Charles P. Vega, MD, has disclosed the following relevant financial relationships: Served as an advisor or consultant for: Lundbeck, Inc; McNeil Pharmaceuticals; Takeda Pharmaceuticals North America, Inc.

**AUTHORS AND CREDENTIALS**

Barbara A. Dennison, MD, New York State Department of Health, Albany, New York; School of Public Health, University at Albany — State University of New York, Rensselaer, New York

Disclosure: Barbara A. Dennison, MD, has disclosed no relevant financial relationships.

Bethany Hawke, MPH, Formerly at the New York State Department of Health; currently a medical student at Albany Medical School, Albany, New York

Disclosure: Bethany Hawke, MPH, has disclosed no relevant financial relationships.

Rachael A. Ruberto, MPH, CPH, New York State Department of Health, Albany, New York

Disclosure: Rachael A. Ruberto, MPH, CPH, has disclosed no relevant financial relationships.

Deborah J. Gregg, MPH, RDN, CDN, CLC, New York State Department of Health, Albany, New York

Disclosure: Deborah J. Gregg, MPH, RDN, CDN, CLC, has disclosed no relevant financial relationships.

## Introduction

Breastfeeding provides many health and economic benefits to mothers, infants, and the public (1,2). Hospital maternity care and infant nutrition policies and practices have a profound effect on the breastfeeding success of mothers and infants from their first few days in the hospital through the first 6 months of life (3–6). The World Health Organization (WHO) has identified a set of evidence-based maternity care policies and practices, “Ten Steps to Successful Breastfeeding” (Ten Steps), which have been shown to increase breastfeeding initiation, exclusivity (without supplemental bottle feeding), and duration (3). As the benefits of exclusive breastfeeding for the first 6 months are increasingly recognized, breastfeeding goals have shifted to focus on improvement in hospital maternity care policies and practices to support the establishment, success, and continuation of exclusive breastfeeding for 6 months (1). Exclusive breastfeeding is included in the Joint Commission’s Perinatal Care Core Measures (7), and the US Department of Health and Human Services has increased the *Healthy People 2020* target for exclusive breastfeeding (8).

Incorporating recommended practices into formal written policy is recommended as a first action in instituting organizational and systems changes (9). Written hospital breastfeeding policies are one of the strongest determinants of whether recommended maternity care practices are implemented in hospitals (6) and are associated with a higher prevalence of breastfeeding initiation (6) and longer duration of breastfeeding (10). However, hospitals in the United States, including those in New York State, have been slow to adopt policies and strategies to ensure that WHO’s Ten Steps are incorporated into maternity care practices (9). In 2005, New York Codes, Rules, and Regulations (NYCRR), Title 10, § 405.21 – Perinatal Services, were amended to require hospitals to have a written breastfeeding policy that specifies that services and practices consistent with Ten Steps were to be provided to support women to successfully breastfeed (11,12). Despite these state regulations, a 2009 survey by the Centers for Disease Control and Prevention of New York hospitals showed that only 26% had comprehensive breastfeeding policies (13).

To codify and strengthen New York’s hospital perinatal regulations, New York Public Health Law, Article 25, Title 1, § 2505–a, the Breastfeeding Mothers’ Bill of Rights (BFMBR), was passed in August 2009 with an implementation date of May 2010 (14). The law mandated that each hospital that provides maternity services post a copy of the BFMBR in a public space, provide a copy of the BFMBR to pregnant patients who prebooked or were admitted, and provide a contact number for women who believed they were not provided information on any of the practices.

The New York State health commissioner issued the Call to Action to Promote Breastfeeding, which was designed to increase awareness of the health benefits and public health importance of breastfeeding. In addition, under Public Health Law 2803-j (15), the New York State health commissioner informed hospitals that hospital-specific performance measures for infant feeding and breastfeeding would be added to the maternity information currently required in the *Maternity Information* brochure that must be provided to expectant mothers when they attend prenatal classes, register, or are admitted to a New York hospital. A press release for the Call to Action to Promote Breastfeeding was issued on August 6, 2009 (16) and sent to hospital leaders, directors of maternity services, chiefs of neonatology, chiefs of obstetrics, and breastfeeding coordinators.

On December 13, 2010, and continuing yearly thereafter, the first hospital-specific breastfeeding measures for healthy newborns during the birth hospitalization (based on New York State birth certificate data) were developed, released to the public, and posted on the Department’s website (17). For these purposes, healthy newborns exclude infants who are admitted to a neonatal intensive care unit (NICU) or transferred in or out of the hospital (18). New York hospitals are ranked against each other and against the national *Healthy People 2020* target for the percentage of infants who initiate breastfeeding (ie, are fed any breast milk). In addition, New York has developed 2 additional measures and targets: 1) the percentage of infants fed breast milk exclusively (70% or more), and 2) among breast-fed infants, the percentage who are supplemented with formula (no more than 14.2%).

From 2010 through 2011, the *New York State Model Hospital Breastfeeding Policy and Implementation Guide* was developed, posted, and disseminated (19,20). During February 2011, training webinars designed to increase awareness, knowledge, and understanding of the rationale for the recommended policies and maternity care practices were conducted 9 times for hospital providers and staff and made available on the New York State Department of Health (NYSDOH) website (21).

The purpose of this study was to assess the first stage of incorporating criteria of the BFMBR law into hospital breastfeeding policies. A secondary objective is to describe the potential role of public health activities (education and staff training) and monitoring and enforcement.

## Methods

In fall 2009, 2011, and 2013, NYSDOH informed each New York hospital that provided maternity care that they were required under NYCRR to have a written hospital breastfeeding policy (11), and hospitals were asked to provide a copy of the hospital’s policies and procedures for feeding newborn infants and for supporting breastfeeding. This request was made jointly by senior NYSDOH staff from the Office of Public Health, Division of Chronic Disease Prevention, and from the Office of Health Systems Management, Division of Certification and Surveillance. The requests were sent by email to each hospital’s chief executive officer, director of maternity services, chief of neonatology, chief of obstetrics, and breastfeeding coordinator. NYSDOH followed up with email and telephone calls until the hospitals provided information on their breastfeeding policies and procedures.

We developed a tool for reviewing hospital breastfeeding policy to indicate full compliance with the 28 components of breastfeeding support specified in New York’s hospital regulations or legislation (11,14). This tool was reviewed by lawyers and by senior staff in the NYSDOH Hospital Regulatory Office to ensure it was consistent with the NYCRR and by the Division of Legal Affairs to ensure it was consistent with the BFMBR. Step 1 of Ten Steps, “Have a written hospital breastfeeding policy,” was not included as a measured item in the policy review tool because all New York hospitals had a written breastfeeding policy, and hospitals without a breastfeeding policy would have received a total score of 0 because they would not have had any components to be reviewed. Individual components that aligned with Ten Steps (3) were used to measure whether the hospital included each of the steps in Ten Steps (Table 1).

**Table 1:** New York Hospitals (N = 129) with Policies Incorporating the World Health Organization’s (WHO’s) Recommended 10 Steps to Successful Breastfeeding, 2009–2013

WHO Recommendation	New York Policy Component^a^ Addressing WHO Recommendation	2009, N (%)	2011, N (%)	2013, N (%)	*P* Value^b^
1. Have a written breastfeeding policy that is routinely communicated to all health care staff.	NA	129 (100)	129 (100)	129 (100)	NA
2. Train all health care staff in skills necessary to implement this policy.	1	42 (32.6)	76 (58.9)	125 (96.9)	<.001
3. Inform all pregnant women about the benefits and management of breastfeeding.	3, 4, 5	0 (0.0)	17 (13.2)	117 (90.7)	<.001
4. Help mothers initiate breastfeeding within half an hour of birth.	9	116 (89.9)	128 (99.2)	129 (100)	<.001
5. Show mothers how to breastfeed, and how to maintain lactation even if they should be separated from their infants.	10, 17	27 (20.9)	66 (51.2)	126 (97.7)	<.001
6. Give newborn infants no food or drink other than breast milk, unless medically indicated.	19	115 (89.2)	120 (93.0)	129 (100)	<.001
7. Practice rooming-in; allow mothers and infants to remain together 24 hours a day.	14	108 (83.7)	123 (95.4)	129 (100)	<.001
8. Encourage breastfeeding on demand.	13	123 (95.4)	128 (99.2)	129 (100)	<.01
9. Give no artificial teats or pacifiers (also called dummies or soothers) to breast-fed infants.	21, 22	22 (17.1)	58 (45.0)	126 (97.7)	<.001
10. Foster the establishment of breastfeeding support groups and refer mothers to them on discharge from the hospital or clinic.	23	82 (63.6)	111 (86.1)	129 (100)	<.001
Includes components compliant with all 10 steps	All listed components	0 (0)	11 (8.5)	112 (86.8)	<.001

Abbreviations: NA, not applicable.

a See Table 2 for list of numbered components.

b *P* value reflects Cochran-Armitage test for trend among binomial proportions by year.

In each of the 3 years studied, one reviewer (B.A.H.) used the policy review tool to review and score hospital breastfeeding policies; each of the 28 components of breastfeeding support was coded as present or absent. To be marked present, the entire component needed to be present; no partial credit was given. The sum of the number of items recorded as present equaled the total score with a maximum score of 28.

Following the 2009 and 2011 policy reviews, NYSDOH sent hospital leaders a summary of their hospital’s breastfeeding policy review, noting which of the required components were present or missing, their hospital’s total score based on the number of required components present, and the average total score of all New York hospitals. Hospitals whose written policies did not include all components were asked to revise their breastfeeding policies and procedures and informed that they would be reviewed again for compliance. A follow-up letter sent in October 2012, before the collection of policies in 2013, included stronger regulatory language. Hospitals whose written hospital breastfeeding policy and procedures did not include at least 27 of the required 28 components were informed that they were out of compliance with NYCRR, Title 10, 405.21 – Perinatal Services, or New York Public Health Law, Article 25, Title 1, § 2505-a (BFMBR). Noncomplying hospitals were given a 4-month deadline to revise and resubmit their hospital’s policy to ensure compliance with New York legislation and regulations; they were told that if NYSDOH did not receive a revised and approved written hospital breastfeeding policy by a specified date, a statement of deficiency would be issued and a citation written and transmitted to the hospital. In addition, the hospital would need to provide a plan of correction to NYSDOH for review. Hospitals unwilling or unable to submit an acceptable plan of correction and a hospital breastfeeding policy would be fined.

From 2009 through 2013, due to closure or merger with other hospitals, the number of New York hospitals providing maternity care decreased from 132 to 129. These analyses were therefore limited to the 129 hospitals that provided maternity care services in all years from 2009 through 2013. In 2009, 2011, and 2013, each hospital provided a copy of its written policies and procedures related to infant feeding and breastfeeding support.

For each hospital contacted (n = 129), the total breastfeeding policy score was determined by summing the number of the 28 state-required components present in the hospital’s written breastfeeding policy. Changes in the hospital’s total scores from 2009 through 2011 and from 2011 through 2013 were calculated using PROC GLM (SAS Institute, Inc). Changes in the mean total score from 2009 through 2011 were compared with the changes from 2011 through 2013 by using a paired *t* test. The number and percentage of hospitals with breastfeeding policies that included all of the required 28 components were calculated for 2009, 2011, and 2013. We used the Cochran-Armitage test for trend to assess changes from 2009 through 2011 and from 2011 through 2013 in the proportion of hospitals with breastfeeding policies that included all required components and all of the Ten Steps.

In 2013, a random sample of hospitals was selected to test the 2-rater reliability of the policy coding. In a sample with 70% prevalence, a sample size of 50 would have 80% power to detect a κ of .40 or greater (2-sided test, *P* < .05) (22). Hospital policies were scored independently by 2 reviewers (B.A.H. and D.J.G.). Scores were compared to determine the percentage agreement and interrater reliability by using the κ statistic.

All data analyses were performed with SAS, version 9.3 (SAS Institute, Inc). A 2-sided *P* < .05 was considered significant.

## Results

The total mean policy component score, increased significantly from 10.4 in 2009 to 16.8 in 2011 and to 27.1 in 2013 (*F* = 706.15, *P* < .001). The number and percentage of hospitals that had a fully compliant hospital breastfeeding policy (total score = 28) increased 15-fold during this time period, from 0 in 2009 to 6 (5%) in 2011 to 97 (75%) in 2013(Figure). The increase in the total mean score was 61% greater from 2011 through 2013 than from 2009 through 2011 (*t* = −5.83, *P* < .001). Table 2 shows, for each separate component, how many hospital breastfeeding policies included each component in each of the 3 years. The proportion of hospitals with breastfeeding policies that included all required 28 components increased significantly for each of the components.

**Figure:**
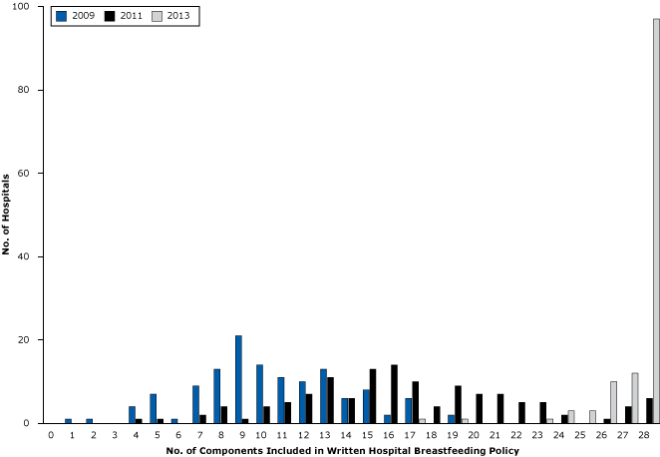
Distribution of New York hospitals (N = 129) that provided maternity services by the total number of state-required components included in each hospital’s written breastfeeding policy in 2009, 2011, and 2013. **Table:** 

No. of Required Components Included	2009	2011	2013
1	1	0	0
2	1	0	0
3	0	0	0
4	4	1	0
5	7	1	0
6	1	0	0
7	9	2	0
8	13	4	0
9	21	1	0
10	14	4	0
11	11	5	0
12	10	7	0
13	13	11	0
14	6	6	0
15	8	13	0
16	2	14	0
17	6	10	1
18	0	4	0
19	2	9	1
20	0	7	0
21	0	7	0
22	0	5	0
23	0	5	1
24	0	2	3
25	0	0	3
26	0	1	10
27	0	4	12
28	0	6	97

**Table 2:** Number of New York Hospitals (N = 129) with Breastfeeding Policies That Include Each of 28 State-Required Components, 2009–2013

Component	2009 N (%)	2011 N (%)	2013 N (%)	*P^a^*
Has a written breastfeeding policy	129 (100)	129 (100)	129 (100)	NA
1. Designate staff to implement breastfeeding policy	42 (32.6)	76 (58.9)	125 (96.9)	<.001
2. Instruct mother on self-care and infant care	3 (2.3)	23 (17.8)	121 (93.8)	<.001
3. Educate mother about breastfeeding	4 (3.1)	44 (34.1)	120 (93.0)	<.001
4. Provide mother with complete information on benefits of breastfeeding	46 (35.7)	103 (79.8)	128 (99.2)	<.001
5. Ensure that education is commercial-free	4 (3.1)	24 (18.6)	123 (95.4)	<.001
6. Make no standing orders made for antilactation drugs	30 (23.3)	74 (57.4)	124 (96.1)	<.001
7. Provide information on drugs that may dry up breast milk	17 (13.2)	69 (53.5)	123 (95.4)	<.001
8. Keep infant with mother after birth	82 (63.6)	112 (86.8)	129 (100)	<.001
9. Initiate contact between mother and infant immediately following birth	116 (89.9)	128 (99.2)	129 (100)	<.001
10. Make assistance with breastfeeding always available	38 (29.5)	71 (55.0)	127 (98.5)	<.001
11. Provide mother with information about her progress in breastfeeding	17 (13.2)	48 (37.2)	124 (96.1)	<.001
12. Make available specially trained staff for breastfeeding special-needs infants	52 (40.3)	87 (67.4)	125 (96.9)	<.001
13. Encourage feeding on demand	123 (95.4)	128 (99.2)	129 (100)	<.01
14. Make rooming-in available	108 (83.7)	123 (95.4)	129 (100)	<.001
15. Assure that mothers can breastfeed anytime, day or night	116 (89.9)	129 (100)	129 (100)	<.001
16. Facilitate breastfeeding in the neonatal intensive care unit	14 (10.9)	54 (41.9)	129 (100)	<.001
17. Use mother’s expressed breast milk when mother is unable to nurse	100 (77.5)	116 (89.9)	128 (99.2)	<.001
18. Provide electric breast pumps and rooming-in facilities for re-hospitalized mothers or infants	6 (4.7)	31 (24.0)	121 (93.8)	<.001
19. Restrict supplemental feedings (feedings with infant formula)	115 (89.2)	120 (93.0)	129 (100)	<.001
20. Inform mother if physician has advised against breastfeeding	16 (12.4)	47 (36.4)	127 (98.5)	<.001
21. Enable mother to request no bottle-feeding and a breastfeeding sign on crib	25 (19.4)	62 (48.1)	126 (97.7)	<.001
22. Enable mother to request no pacifiers	72 (55.8)	107 (83.0)	129 (100)	<.001
23. Provide information on breastfeeding resources in the community	82 (63.6)	111 (86.1)	129 (100)	<.001
24. Provide information about available pediatric health-care providers and the importance of follow-up	3 (2.3)	22 (17.1)	128 (99.2)	<.001
25. Ascertain that mothers can perform basic self-care and infant care before discharge or provide additional instruction in self-care	8 (6.2)	45 (34.9)	123 (95.4)	<.001
26. Instruct and counsel about family planning	5 (3.9)	27 (20.9)	122 (94.6)	<.001
27. Inform mother about importance of follow-up care within timeframe as directed by pediatric care provider	40 (31.0)	87 (67.4)	127 (98.5)	<.001
28. Restrict provision of discharge packs containing infant formula or formula coupons	58 (45.0)	96 (74.4)	125 (96.9)	<.001

Abbreviation: NA, not applicable.

a *P* value reflects Cochran-Armitage test for trend among binomial proportions, by year.

The number of hospital breastfeeding policies that included steps 2 through 10 of WHO’s Ten Steps or all 10 steps increased significantly from 2009 through 2013 (Table 1).

Among the random sample (n = 46) of 2013 hospital breastfeeding policies that were independently coded by the 2 reviewers, the percentage agreement was high (*r* = 95%). The interrater reliability among the coders was fair (κ = 0.27) (22).

## Discussion

This study described the implementation of a statewide policy requiring evidence-based support for breastfeeding in maternity hospitals, the state’s monitoring and enforcement activities, and subsequent compliance of hospitals with the components of New York legislation and regulations. Results suggest that legislation, promotion, and education can moderately improve adoption of best-practice policies; however, active monitoring and enforcement may be necessary for broad adoption.

New York was the first state to adopt legislation that required hospitals to ensure that women and their newborns received recommended maternity care during a perinatal admission. This legislation was adopted in response to concerns that women were not receiving recommended maternity care and that hospital staff members were not aware of their shortcomings in support for breastfeeding success (23).

State public health departments can provide technical assistance and guidance as well as regulatory authority to monitor and enforce compliance with policies. Following initial passage of BFMBR in 2009, educational activities and assessment of hospital policies were focused on providing information and individual feedback for quality improvement purposes. This was associated with a small, but significant, improvement in hospital policies; however, most hospitals still had many deficiencies, and their policies did not include all 10 of WHO’s Ten Steps. Marked improvement was seen after the second policy review when there was a shift to a stricter regulatory approach threatening enforcement actions, including potential fines. From 2011 through 2013, full compliance improved 15-fold, from 5% to 75%.

Studies suggest that increased monitoring and enforcement of regulations and laws result in improved compliance and greater impact (24). Examples include the decline in adolescent smoking that was directly associated with the degree of local and state enforcement of laws prohibiting tobacco sales to minors (25). Another example is New York State’s regulation limiting the number of hours that medical residents can work (26). In both cases, compliance was low until stricter enforcement, inspections, and fines were put in place (27).

Previous research has shown that state laws protecting and supporting breastfeeding in the workplace affected breastfeeding outcomes. States that adopted laws requiring employers to provide break time and private space for breastfeeding mothers to pump breast milk had greater increases in breastfeeding initiation and duration than states that did not implement such laws (28).

This study has several limitations. Our findings are observational. The passage of the new legislation (BFMBR) was accompanied by increased monitoring, stricter regulatory enforcement, and the threat of fines. This combination led to increased hospital compliance with breastfeeding policies. In addition, the educational and advocacy activities associated with efforts to gain legislative support and the activities following the passage of this new state law may have contributed to the changes in hospital breastfeeding policies. It may be that as in other areas of public health, such as tobacco-control efforts, multiple strategies and actions across socioecological levels (eg, government, institutions, intrapersonal, individual) are necessary to change the political, administrative, and societal norms for maternity care practices and policies that support successful breastfeeding, both in the hospital and beyond the hospital setting.

This study was not designed to assess whether or not hospitals’ written policies were being followed. Implementation of policy changes may require staff training, education, and skills development and organizational change, institutional buy-in, and community and family support to improve maternity care and breastfeeding outcomes. The impact of the education or training activities on knowledge or attitudes of staff, physicians, or administrators, and whether education and training facilitated policy change, could not be assessed or separately evaluated in this study.

Our study, however, has numerous strengths. First, all New York hospitals that provide maternity care services submitted their written breastfeeding policies in each of the 3 years, 2009, 2011 and 2013. A hospital breastfeeding policy review tool was created, and written hospital breastfeeding policies were objectively reviewed with the same review tool each year. This provided objective measures of hospital policy change over time. In addition, there was a high level of hospital compliance with all required policy components (75%) in 2013, which contributed to a lower interrater reliability κ value (22).

In 2013, 97 of 129 hospitals had a fully compliant hospital breastfeeding policy. Since then, additional individual feedback was provided to the 32 noncompliant hospitals to resolve missing required policy components, and each hospital submitted a revised policy. As of April 2014, all 129 hospitals in New York had a fully compliant, approved, written breastfeeding policy in place.

Further research is needed to determine the impact of improved hospital breastfeeding policies on maternity care practices and on breastfeeding outcomes, such as prevalence of breastfeeding initiation and exclusive breastfeeding in the hospital. In addition, long-term studies are needed to assess the impact of breastfeeding policies on the duration of breastfeeding and on infant, child, and maternal health outcomes.

## Figures and Tables

**Table:** 

**1. The activity supported the learning objectives.**				
**Strongly Disagree**				**Strongly Agree**
1	2	3	4	5
**2. The material was organized clearly for learning to occur.**				
**Strongly Disagree**				**Strongly Agree**
1	2	3	4	5
**3. The content learned from this activity will impact my practice.**				
**Strongly Disagree**				**Strongly Agree**
1	2	3	4	5
**4. The activity was presented objectively and free of commercial bias.**				
**Strongly Disagree**				**Strongly Agree**
1	2	3	4	5

## Post-Test Questions

### Article Title: Implementing Legislation to Improve Hospital Support of Breastfeeding, New York State, 2009–2013

**CME Questions**

1. Which of the following statements regarding the regulation of breastfeeding practices in New York hospitals described in the current study is ***most*** accurate?
  - Hospitals were required to provide breastfeeding information only to expecting mothers who asked for this information
  - There has been no mechanism to report on hospital-specific data on breastfeeding
  - New York has set a target that 95% of infants be initiated exclusively on breast milk
  - Breastfeeding policies do not apply to newborns in the neonatal intensive care unit
2. At the time of the passage of legislation in 2009, what percentage of New York hospitals met all 10 criteria of the World Health Organizations’s ***Ten Steps to Successful Breastfeeding***?
  - 0%
  - 12%
  - 38%
  - 55%
3. Which of the following effects was noted after the regulation of breastfeeding promotion in the current study?
  - Increase in the number of components in breastfeeding policies, but no change in compliance to policies
  - Simplification of breastfeeding policy components, with a subsequent increase in compliance
  - More than doubling in the number of components of breastfeeding policies plus a large increase in compliance to policies
  - No significant change in hospital policy or practice regarding breastfeeding
4. Which of the following statements regarding the process of change of hospital breastfeeding policies in the current study is ***most*** accurate?
  - Passage of the legislation itself was the most critical element in improving compliance with breastfeeding promotion protocols
  - Most of the improvement in adherence to breastfeeding promotion was noted during the period of formal regulatory enforcement
  - Only releasing breastfeeding data to the public prompted changes in hospital protocols
  - Significant improvements in breastfeeding promotion protocols were occurring regardless of regulatory changes

**Evaluation**
